# Validation of cardiovascular outcomes and risk factors in the Clinical Practice Research Datalink in the United Kingdom

**DOI:** 10.1002/pds.5150

**Published:** 2020-10-28

**Authors:** Alejandro Arana, Andrea V Margulis, Cristina Varas‐Lorenzo, Christine L Bui, Alicia Gilsenan, Lisa J McQuay, Maria Reynolds, Cristina Rebordosa, Billy Franks, Stefan de Vogel, Kwame Appenteng, Susana Perez‐Gutthann

**Affiliations:** ^1^ Pharmacoepidemiology and Risk Management RTI Health Solutions Barcelona Spain; ^2^ Pharmacoepidemiology and Risk Management RTI Health Solutions Research Triangle Park North Carolina USA; ^3^ Biostatistics RTI Health Solutions Research Triangle Park North Carolina USA; ^4^ Astellas Leiden Netherlands; ^5^ Astellas Northbrook Illinois USA

**Keywords:** acute myocardial infarction, electronic record, information bias, menopause, obesity, pharmacoepidemiology, smoking, stroke, validity

## Abstract

**Purpose:**

Strategies to identify and validate acute myocardial infarction (AMI) and stroke in primary‐care electronic records may impact effect measures, but to an unknown extent. Additionally, the validity of cardiovascular risk factors that could act as confounders in studies on those endpoints has not been thoroughly assessed in the United Kingdom Clinical Practice Research Datalink's (CPRD's) GOLD database. We explored the validity of algorithms to identify cardiovascular outcomes and risk factors and evaluated different outcome‐identification strategies using these algorithms for estimation of adjusted incidence rate ratios (IRRs).

**Methods:**

First, we identified AMI, stroke, smoking, obesity, and menopausal status in a cohort treated for overactive bladder by applying computerized algorithms to primary care medical records (2004–2012). We validated these cardiovascular outcomes and risk factors with physician questionnaires (gold standard for this analysis). Second, we estimated IRRs for AMI and stroke using algorithm–identified and questionnaire–confirmed cases, comparing these with IRRs from cases identified through linkage with hospitalization/mortality data (best estimate).

**Results:**

For AMI, the algorithm's positive predictive value (PPV) was >90%. Initial algorithms for stroke performed less well because of inclusion of codes for prevalent stroke; algorithm refinement increased PPV to 80% but decreased sensitivity by 20%. Algorithms for smoking and obesity were considered valid. IRRs based on questionnaire‐confirmed cases only were closer to IRRs estimated from hospitalization/mortality data than IRRs from algorithm‐identified cases.

**Conclusions:**

AMI, stroke, smoking, obesity, and postmenopausal status can be accurately identified in CPRD. Physician questionnaire–validated AMI and stroke cases yield IRRs closest to the best estimate.

**Key Points**

- Algorithms to detect acute myocardial infarction in the Clinical Practice Research Datalink (CPRD) GOLD database performed very well (positive predictive value [PPV] >90%).
- Algorithms for stroke performed less well (PPV = 56%) because they included codes for prevalent stroke. Excluding these codes increased the PPV to 80% but decreased sensitivity by 20%.
- Smoking, obesity, and postmenopausal status can be accurately identified using algorithms that rely on information in the electronic medical records in CPRD GOLD.
- For subjects whose information comes from general practitioners only, incidence rate ratios that used only cases confirmed by physicians via questionnaire were closer to incidence ratios from hospitalization and mortality data (best estimate) than estimates that used all algorithm‐identified cases including cases that were not physician confirmed. This suggests that endpoint validation decreased misclassification bias.

## 1 INTRODUCTION

The usefulness of electronic medical records for epidemiologic research relies on data accuracy. In the United Kingdom, the Clinical Practice Research Datalink (CPRD) contains demographic, clinical, and drug prescription data, constituting a rich source for epidemiological research.^1^, ^2^, ^3^, ^4^ Linkage to hospital and death records, generally considered reliable sources for health events, is possible for a proportion of UK primary care practices, enhancing the validity and utility of the CPRD.^1^

Formerly, confirmation of events in primary care data often relied on physicians' unstructured notes, known as free‐text comments^1^, ^5^; however, free‐text comments from CPRD are no longer available for research. Currently, confirmation of events, treatments, dates, and patient characteristics is possible through questionnaires sent to general practitioners (GPs), a common strategy for researchers to confirm events in the subset of the population for whom linkage to hospital and death records is not possible.

Little research has been conducted to validate patient characteristics that are considered cardiovascular (CV) risk factors and potential confounders for CV outcomes.^6^, ^7^ Moreover, the impact of misclassification of myocardial infarction and stroke on effect measures when using primary care data that cannot be linked to hospital or mortality data has not yet been evaluated. The validation component of a larger drug safety study^8^ provided an opportunity to evaluate the validity of algorithms for identifying CV risk factors from primary care data and to examine the effect of various outcome‐identification strategies reliant on those algorithms when estimating the incidence of CV outcomes.

This article has two parts. In the first part, we explored the validity of algorithms to identify acute myocardial infarction (AMI) and stroke that use only structured data from primary care and explored the accuracy of algorithms for recorded smoking, obesity, and menopause. In the second part, we examined the effect of four outcome‐identification strategies, applying our validated algorithms, on the estimation of adjusted incidence rate ratios (IRRs) for AMI and stroke.

## 2 METHODS

### 2.1 Setting

This study was the validation component of a drug safety study^8^ (“parent study”) that included patients aged ≥18 years who were continuously enrolled in the UK CPRD primary care database (GOLD) for ≥12 months and were newly exposed to antimuscarinic medications to treat overactive bladder (darifenacin, fesoterodine, oxybutynin, solifenacin, tolterodine, or trospium) in 2004 to 2012. Patients with a diagnosis of cancer other than nonmelanoma skin cancer were excluded, as were HIV+ patients because their health service utilization might not be fully captured. Patients were followed until death, disenrollment, cancer diagnosis, HIV+ status, AMI, stroke, or study end, whichever occurred first.

### 2.2 Data source

This study used information collected in the CPRD.^1^ CPRD GOLD contains pseudonymized electronic medical records for 11.3 million people.^1^ Data include diagnoses, symptoms, referrals, tests, prescriptions issued, and additional clinical information. Drugs are classified following the British National Formulary, and medical data are coded according to the Read coding system. The latter is very granular and is regularly updated in response to physician user requests.

CPRD GOLD data can be linked with Hospital Episode Statistics Admitted Patient Care (HES APC) data and the Office for National Statistics (ONS) mortality data. HES APC data include admission and discharge dates and diagnoses of all hospitalizations and are coded according to the *International Classification of Diseases, 10th Revision* (ICD‐10). ONS data include date of death, place of death, underlying cause of death, and all other causes of death listed on the death certificate, also coded according to ICD‐10.

In our cohort of patients treated for overactive bladder, CV outcomes and mortality were ascertained from general practice records in the CPRD GOLD and via linkage to HES APC data and ONS mortality data for the subset of general practices that permit such linkage.^1^ For this study, we divided the study population into two groups: subjects with information from general practice records only (“CPRD unlinked”) and subjects whose records from general practices could be linked to external data sources, including hospitalizations and mortality data (“CPRD linked”). Approximately 50% of the cohort were in the CPRD‐unlinked group, and approximately 50% were in the CPRD‐linked group.

### 2.3 Part 1. Validation of CV Outcomes and Risk Factors

#### 2.3.1 Study Participants

Validation was conducted within a subset of CPRD‐unlinked data from the parent study. This subset comprised all patients with prescriptions for the least commonly prescribed drugs (darifenacin, fesoterodine, and trospium) and a randomly selected one‐third of the patients with a prescription for each of the most commonly prescribed drugs (oxybutynin, solifenacin, and tolterodine).^10^

#### 2.3.2 Algorithms to ascertain CV Events and risk factors

The CV events validated in this study were AMI and stroke (separately for ischemic, hemorrhagic, and unspecified stroke). The CV risk factors validated in this study were smoking (never/current/former smoker), obesity (yes/no, defined as body mass index≥30 kg/m^2^), and menopause (yes/no).

Each computerized algorithm, including code lists and processes to identify and use those code lists, was designed by a cardiologist epidemiologist with experience in CV research in CPRD and other data sources (C.V.L.) and discussed with two other physician epidemiologists (A.A., A.V.M.), taking into consideration, among other aspects, code use frequency. We used algorithms to search electronic medical records for codes for diagnoses, signs, symptoms, laboratory tests, prescriptions, specialist referrals, hospitalizations, and structured data elements, known as *entities* that provide additional clinical or other details that are not stored as coded data in GOLD, such as the number of cigarettes smoked per day. All code lists are available in Supporting Information (Code Lists‐SuppInfo).

The computerized algorithms for AMI and stroke were based on clinical definitions for each type of event.^11^, ^12^ Out‐of‐hospital deaths due to these CV events can be difficult to identify in electronic health care data; our algorithms were designed to identify these events as completely as possible (Table 1).

**TABLE 1:** Definitions to identify and classify AMI and stroke

**AMI**
**Definite AMI:** A Read code for AMI with hospitalization AND two or more Read codes for the following events within 30 days before or after the AMI code: Characteristic chest pain or symptoms of myocardial ischemia^a^ Abnormal results for cardiac enzymes^a^ Electrocardiogram with clinical signs of AMI^a^ Arteriogram with a recent coronary occlusion^a^ Administration of thrombolytic therapy^a^ Coronary revascularization procedure^a^ following AMI diagnosis Death
**Probable AMI:** A Read code for AMI with hospitalization and/or one item above within 30 days before or after the AMI code
**Possible AMI:** A Read code for AMI but no code for any of the items above within 30 days of the AMI code
**Stroke**
**Definite stroke:** A Read code for stroke (not including transient ischemic attack) and two or more codes for the following events (within 30 days before or after the stroke code): Diagnostic procedure with abnormal results Hospitalization or referral to a neurologist Acute treatment Residual damage Physiotherapy Death
**Probable stroke:** A stroke code and one of items listed above within 30 days before or after the stroke code
**Possible stroke:** A stroke code and none of the items listed above

Abbreviation: AMI, acute myocardial infarction.

^a^ Considered a sign or symptom of AMI.

The AMI and stroke algorithms used combinations of coded entries in the appropriate time windows to identify AMI and stroke events and categorize them as definite, probable, or possible cases (details on the combinations of entries and time windows are provided in Table 1). Patients lacking a specific AMI diagnosis but with the signs and symptoms of AMI listed in Table 1 were categorized as having a potential AMI. Other individuals were considered noncases, further subclassified as noncases alive (if they were alive at the end of the study period) or noncases dead (if their death occurred during the study period). Death and CV outcomes in these patients were ascertained from CPRD GOLD data for the CPRD‐unlinked population.

The algorithms for smoking, obesity, and menopause were developed collaboratively by the three physician epidemiologists and other coauthors of the study (Table 2). For smoking, a key consideration was that a record indicating that a patient was a smoker was given more weight than a record indicating that a patient had never smoked, under the assumption that the smoking habit (recorded in the electronic medical record) might have triggered a medical intervention. Patients with early codes for smoking and more recent codes for not smoking were considered former smokers. The algorithm for obesity was designed to capture the most recent information in the medical record; weight and height measurements were preferred over codes for obesity. Smoking, obesity, and menopause were ascertained at three time points using all information available: the day before cohort entry, the last date with information before the CV outcome, and the date with information closest to the outcome (before or after). Validation was performed for ascertainment at each of the three dates, for comparison. To enhance the ascertainment of postmenopausal status, we conducted a post‐hoc analysis of an algorithm to identify menopause that added two proxies for postmenopausal status: (patients aged >50 years who used HRT therapy and patients aged >55 years) to the original algorithm.

**TABLE 2:** Algorithms to define smoking, obesity, and postmenopausal status

Covariate	Definition
Smoking	Defined based on Read code entries or entities (entity 4) If most recent code is for current smoker ➔ current smoker If most recent code is for former smoker ➔ former smoker If most recent code is for never smoker: If a prior code is for current or former smoker ➔ former smoker Otherwise ➔ never smoker
Obesity	Order all entity entries within the appropriate time frame (3 years if possible) for weight or height (entities 13 and 14, respectively). Find the most recent entry for entity 14. Data field 3 is BMI. If data field 3 is empty, find the most recent entry for height. Calculate BMI as weight in kilograms (entity 13, data field 1) divided by the square of height in meters (entity 14). Categorize BMI: BMI < 30 ➔ not obese BMI ≥30 ➔ obese If missing, complete with information from Read codes for obesity.
Postmenopausal status	Defined based on Read code entries for menopause.

Abbreviation: BMI, body mass index.

#### 2.3.3 Questionnaires

Validation of CV outcomes and risk factors was conducted by comparing the classification reached by using algorithms with that attained by using questionnaires (the gold standard for validation analyses) sent to GPs of the patients identified by the algorithms for the following groups of cases:

- All definite and **possible AMI** and **stroke** cases
- All **potential AMI** cases
- 185 randomly selected patients in each of the event categories **probable AMI**, **probable stroke**, **noncase dead**, and **noncase alive**; the goal was to obtain 150 responses with an expected 80% response rate

In the questionnaires (see Supporting Information: Questionnaires‐SuppInfo), GPs confirmed the outcome and reported on patients' smoking history, obesity, and menopausal status at the time of the AMI or stroke or at study end in noncases alive or dead.

Positive predictive values (PPVs) were estimated as the probability that a subject identified as a case by the algorithm was confirmed as such via questionnaire response. The negative predictive value (NPV) was defined as the probability that a subject classified as a noncase remained as such via questionnaire response. For AMI, stroke, obesity, and menopausal status, we estimated PPVs and NPVs. For smoking history, we assessed whether records from our algorithms were in concordance with responses in physician questionnaires (ie, algorithm‐defined current smoking would be considered concordant with a questionnaire response indicating current smoker status).

### 2.4 Part 2. Understanding outcome misclassification

As a separate research question and to understand the impact of outcome misclassification on a safety question, we compared the IRRs of four outcome‐identification strategies using primary care data in the CPRD‐unlinked population with the IRRs estimated using primary care, HES APC, and ONS data in the CPRD‐linked population.

#### 2.4.1 Study participants

The four outcome‐identification strategies were applied to the study population that was included in Part 1 of this study (CPRD‐unlinked population). In addition, we used the CPRD‐linked population from the parent study for comparison.

#### 2.4.2 Outcome‐identification strategies and estimation of IRRs

We estimated propensity score‐adjusted IRRs of AMI and stroke for oxybutynin (commonly used to treat overactive bladder and has been on the market for many years) vs any other study medication using four outcome‐identification strategies that made use of the algorithms and validation efforts described in Part 1 of this manuscript, separately for AMI and stroke:

- Strategy A: all cases confirmed through questionnaires (most stringent strategy)
- Strategy B: cases identified in strategy A, plus cases that could not be evaluated through questionnaires (eg, questionnaires were not returned)
- Strategy C: all electronically identified cases (no validation by GP)
- Strategy D (AMI only): cases identified in strategy C plus potential AMI cases (most lenient strategy, more sensitive definition)

The propensity score methods used for this analysis have been previously described.^13^ The IRRs estimated using these strategies were compared with IRRs estimated from cases identified in the CPRD‐linked population (“best estimate”).

We plotted the *P* value functions^14^ of the estimated IRR for these four strategies to summarize two key aspects of this analysis: the estimated effect size (represented by the horizontal location of the peak of each curve) and the degree to which the various strategies were compatible (indicated by the proportion of possible overlap of the area under the curves).

## 3 RESULTS

### 3.1 Part 1. Validation of CV Outcomes and risk factors

The parent study cohort consisted of 119 912 patients, 70% of whom were women. Mean ages at cohort entry were 64.5 years (men) and 61.5 years (women). Among the 26 511 participants in Part 1 of this validation study, the algorithm identified a total of 2658 AMIs and 726 strokes in primary care data.

#### 3.1.1 Questionnaires

In the validation study, 2339 questionnaires were sent to GPs. The proportions of questionnaire response were similar across endpoints: AMI, 80%; stroke, 83%; noncase alive, 81%; and noncase dead, 79% (Table 3).

**TABLE 3:** Results of validation of AMI and stroke events^a^

	Questionnaires sent	Questionnaires returned, response rate (%)	Case status confirmed by questionnaire	PPV, % (95% CI)	NPV, % (95% CI)
**Decision from original algorithm**					
AMI					
Definite	137	114 (83)	112	98 (94‐100)	
Probable	162	134 (83)	123	92 (86‐96)	
Possible	32	24 (75)	22	92 (73‐99)	
Signs and symptoms only	1097	864 (79)	22	2.5 (1.6‐3.8)	
Stroke—original definition					
Definite	157	131 (83)	101	77 (69‐84)	
Ischemic	84	69 (82)	64	93 (84‐98)	
Hemorrhagic	21	17 (81)	17	100 (80‐100)	
Unspecified	52	45 (87)	20	44 (30‐60)	
Probable	249	207 (83)	97	47 (40‐54)	
Ischemic	79	70 (89)	62	89 (79‐95)	
Hemorrhagic	23	22 (96)	16	73 (50‐89)	
Unspecified	147	115 (78)	19	17 (10‐25)	
Possible	135	114 (84)	48	42 (33‐52)	
Ischemic	41	39 (95)	33	85 (69‐94)	
Hemorrhagic	9	9 (100)	8	89 (52‐100)	
Unspecified	85	66 (78)	7	11 (4‐21)	
Noncases alive^b^	185	149 (81)	147		99 (95‐100)
Noncases dead^c^	185	146 (79)	123		84 (77‐90)
**Decision from revised algorithm for stroke**					
Stroke—updated definition					
Definite	120	98 (82)	90	92 (85‐96)	
Ischemic	75	60 (80)	57	95 (86‐99)	
Hemorrhagic	20	16 (80)	16	100 (79‐100)	
Unspecified	25	22 (88)	17	77 (55‐92)	
Probable	107	90 (84)	71	79 (69‐87)	
Ischemic	55	47 (85)	42	89 (77‐96)	
Hemorrhagic	22	21 (95)	15	71 (48‐89)	
Unspecified	30	22 (73)	14	64 (41‐83)	
Possible	48	44 (92)	37	84 (70‐93)	
Ischemic	31	29 (94)	25	86 (68‐96)	
Hemorrhagic	8	8 (100)	7	88 (47‐100)	
Unspecified	9	7 (78)	5	71 (29‐96)	

Abbreviations: AMI, acute myocardial infarction; CI, confidence interval; NPV, negative predictive value; PPV, positive predictive value.

^a^ Responses from questionnaires were the gold standard for this analysis. A total of 2364 questionnaires were initially sent, but only 2339 corresponded to participants who were retained in the study after applying all eligibility criteria. Questionnaires for patients not retained in the study are not included in these results.

^b^ Noncases alive: patients without AMI or stroke in Clinical Practice Research Datalink primary care data who were alive at the end of follow‐up.

^c^ Noncases dead: patients without AMI or stroke in Clinical Practice Research Datalink primary care data who had died by the end of follow‐up.

#### 3.1.2 Validation of AMI and stroke

The algorithm for AMI yielded PPVs >90% for definite, probable, and possible AMI. The PPV for AMI signs and symptoms only was 2.5% (95% confidence interval [CI], 1.6%‐3.8%) (Table 3).

The initial algorithm for incident stroke resulted in low PPVs (Table 3); 56% of strokes were confirmed. Review of medical profiles for unconfirmed cases revealed that four codes used to electronically identify strokes did not clearly represent incident events (Read codes 662 M.00 [stroke monitoring], 662o.00 [hemorrhagic stroke monitoring], 8HTQ.00 [referral to stroke clinic], and 9N0p.00 [seen in stroke clinic]), and most probably reflected prevalent stroke. When these codes were excluded, the updated algorithm yielded much higher PPVs (95% CIs): 92% (85%‐96%) for definite stroke, 79% (69%‐87%) for probable stroke, and 84% (70%‐93%) for possible stroke. However, removing these codes from the algorithm decreased its sensitivity by 20%: of 246 stroke cases identified with the initial algorithm and later confirmed through questionnaires, 48 were missed by the revised algorithm. For ischemic and hemorrhagic stroke, PPVs were high (eg, 93% for definite ischemic stroke and 100% for definite hemorrhagic stroke). While PPVs for ischemic and hemorrhagic stroke were of similar magnitude, PPVs for unspecified stroke were lower. The updated algorithm increased the PPVs, mainly for unspecified strokes.

The algorithm for noncase alive had an NPV of 99% (95% CI, 95%‐100%) (Table 3). The algorithm for noncase dead did not perform as well: 16% were found to have had an AMI or stroke; death was confirmed in all.

#### 3.1.3 Validation of smoking, obesity, and postmenopausal status

Among patients with completed questionnaires (the denominator for concordance), three had information missing on smoking in primary care data. At the date closest to and before the endpoint, 97% of the patients identified as never smokers in primary care data were also never smokers according to questionnaires (Table 4). Likewise, 84% identified as current smokers were also current smokers according to questionnaires, and 77% of former smokers were also former smokers according to questionnaires. Concordance at the three evaluated time points is presented in Table 4.

**TABLE 4:** Concordance between primary care data from the CPRD and information from questionnaires: smoking^a^

CPRD data	Questionnaires			
	Never smoker	Current smoker	Former smoker	Unknown
Before cohort entry				
Never smoker, n (%)	718 (93.5)	6 (0.8)	37 (4.8)	7 (0.9)
Current smoker, n (%)	11 (3.7)	232 (77.1)	52 (17.3)	6 (2.0)
Former smoker, n (%)	111 (17.1)	32 (4.9)	492 (75.6)	16 (2.5)
Unknown, n (%)	7 (63.6)	0	4 (36.4)	0
Last date with information before endpoint				
Never smoker, n (%)	708 (96.7)	3 (0.4)	15 (2.0)	6 (0.8)
Current smoker, n (%)	13 (4.5)	241 (84.0)	27 (9.4)	6 (2.1)
Former smoker, n (%)	123 (17.3)	26 (3.7)	543 (76.6)	17 (2.4)
Unknown, n (%)	3 (100.0)	0	0	0
Date with information closest to the endpoint (before or after)				
Never smoker, n (%)	702 (97.2)	3 (0.4)	11 (1.5)	6 (0.8)
Current smoker, n (%)	15 (5.8)	212 (81.9)	26 (10.0)	6 (2.3)
Former smoker, n (%)	129 (17.2)	55 (7.3)	548 (73.2)	17 (2.3)
Unknown, n (%)	1 (100.0)	0	0	0

Abbreviation: CPRD, Clinical Practice Research Datalink.

^a^ The algorithm identified smoking in primary care data at three time points: the day before cohort entry, the last date with information before the endpoint, and the date with information closest to the endpoint (before or after). General practitioners were asked to provide the same information via questionnaires on the day of the event (patients with AMI or stroke) or at study end (noncases alive or dead). Responses from questionnaires were the gold standard for this analysis. Percentages are row percentages.

**FIGURE 1:**
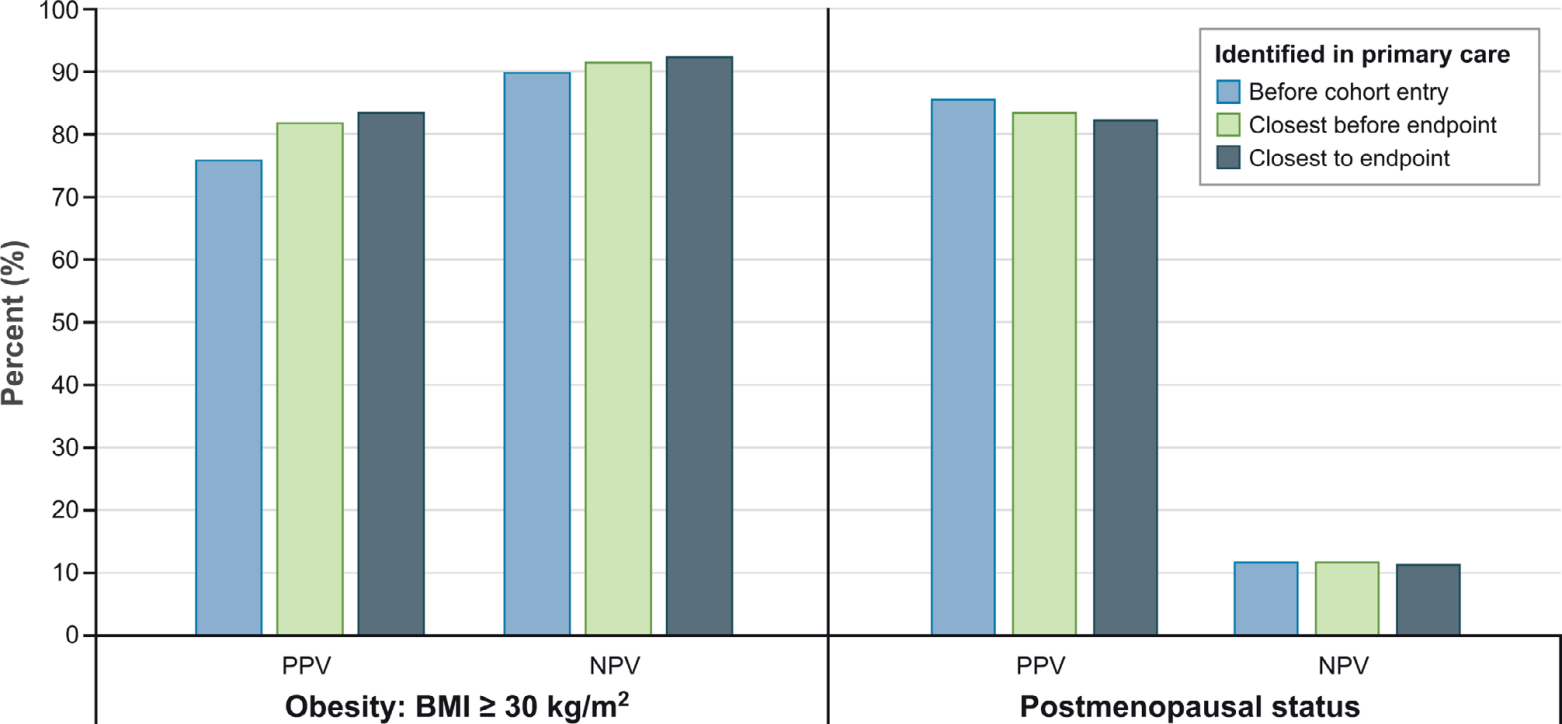
Predictive values of obesity and postmenopausal status. BMI, body mass index; NPV, negative predictive value; PPV, positive predictive value. The algorithm identified patient characteristics in primary care data at three time points: the day before cohort entry, the last date with information before the endpoint, and the date with information closest to the endpoint (before or after). General practitioners were asked to provide the same information via questionnaires on the day of the event (patients with AMI or stroke) or at study end (noncases alive or dead). Questionnaires were the gold standard for this analysis [Colour figure can be viewed at wileyonlinelibrary.com]

Information on obesity was missing in primary care data for 26% of patients on the date closest to and before the endpoint. Of patients classified as obese in primary care data, 82% were confirmed as obese through questionnaires; 92% of patients classified as nonobese were confirmed as nonobese through questionnaires. Among women identified as postmenopausal at baseline in primary care data, 86% were confirmed as such through questionnaires. The algorithm that incorporated proxies for postmenopausal status increased the PPV to 91%. Premenopausal status was confirmed with the initial algorithm in only 12%; this number increased to 47% with the expanded algorithm (Figure 1 and Supporting Information: Table S1‐SuppInfo). The estimated prevalence of postmenopausal status increased from 24% with the initial algorithm to 95% with the expanded algorithm.

### 3.2 Part 2. Understanding outcome misclassification

**FIGURE 2:**
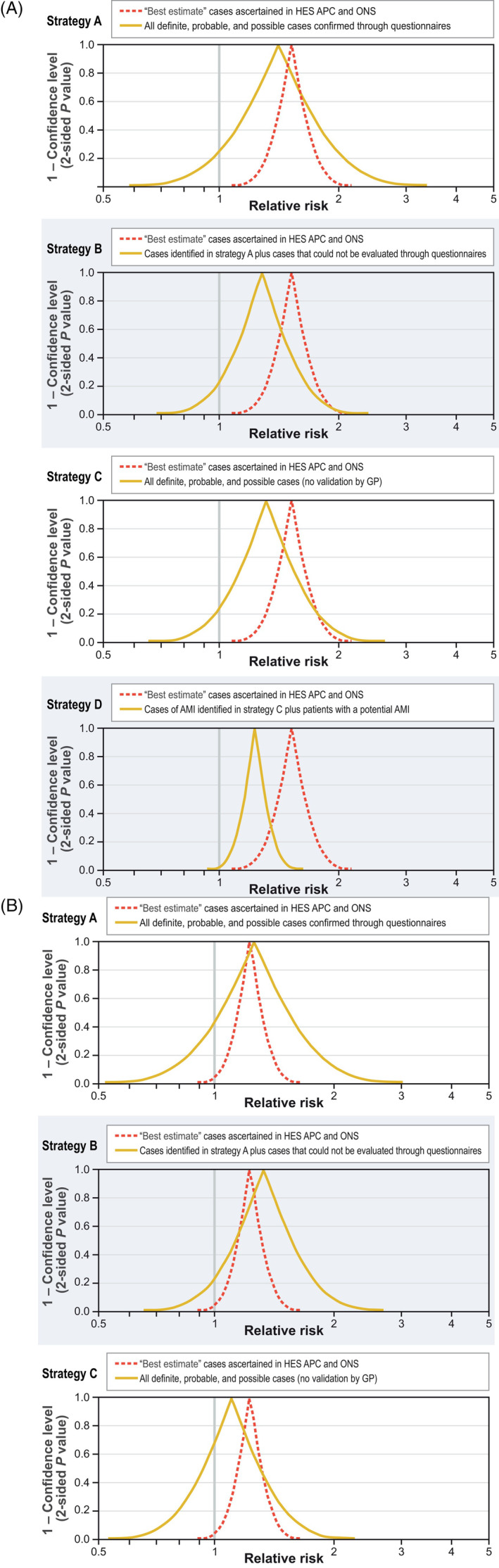
*P* value functions of the incidence rate ratios for AMI and stroke using various outcome‐ascertainment strategies. (A) AMI. (B) Stroke. AMI, acute myocardial infarction; GP, general practitioner; HES APC, Hospital Episode Statistics Admitted Patient Care; ONS, Office for National Statistics [Colour figure can be viewed at wileyonlinelibrary.com]

The most stringent outcome‐identification strategy (strategy A), treating as cases only those that were confirmed through questionnaires, yielded an IRR closer to the best estimates for AMI and stroke in the CPRD‐linked population than the other three strategies (Table 5 and Figure 2). For AMI, the estimated effects for strategy A and the best estimate were very similar (ie, the peaks of the two curves are close to each other; Figure 2A); although less precise, results from strategy A were compatible with best estimate results (ie, the red curve is contained within the yellow curve). Strategies B to D were further from the best estimate in both aspects, with maximal peak separation, sharpest function, and least curve overlap for the less‐stringent strategy D. For stroke (Figure 2B), the IRR for strategy A also was closest to the best estimate and the curve overlap was maximal, but the peaks of the other strategies did not differ substantially from the peak of the best estimate, and the curves for the various strategies partially overlapped.

**TABLE 5:** Incidence rate ratios for various outcome‐ascertainment strategies^a^

Population	Outcome ascertainment	AMI, IRR (95% CI)	Stroke, IRR (95% CI)
Data linkable to hospitalization and death records (CPRD linked)	Ascertained from hospitalization records (Hospital Episode Statistics) and/or death records (Office for National Statistics)	1.52 (1.21‐1.92)	1.22 (1.00‐1.49)
Study participants (CPRD unlinked)	Strategy A: all definite, probable, and possible cases identified by the algorithm confirmed through questionnaires (most stringent strategy)	1.41 (0.79‐2.51)	1.25 (0.70‐2.24)
	Strategy B: cases identified in strategy A, plus cases that could not be evaluated through questionnaires (eg, the questionnaire was not returned or was incomplete)	1.28 (0.85‐1.92)	1.32 (0.83‐2.10)
	Strategy C: All definite, probable, and possible cases identified by the algorithm	1.31 (0.83‐2.07)	1.10 (0.68‐1.76)
	Strategy D: All cases of AMI identified in strategy C, plus patients with AMI signs and symptoms but without an AMI‐specific diagnosis (most lenient strategy)	1.22 (1.02‐1.46)	Not available

Abbreviations: AMI, acute myocardial infarction; CI, confidence interval; IRR, incidence rate ratio.

^a^ IRRs obtained from data linkable to hospitalization and mortality records were the best estimate for this analysis.

## 4 DISCUSSION

The analyses reported here represent real‐world examples of how different strategies can be used to identify and validate CV outcomes and risk factors in primary care data and how the use of these strategies affects IRRs.

We validated CV outcomes and patient characteristics recorded in primary care data, as identified by electronic algorithms, using responses to questionnaires sent to GPs as the gold standard, and we explored the impact of outcome‐identification strategies on the IRR using cases identified from hospital and mortality records as the best estimate. Results of the two evaluations we conducted yield several considerations for future research involving the identification and validation of CV outcomes from CPRD data. First, results from this study support the validity of our definitions for definite, probable, and possible AMI. Our current approach for patients with codes for AMI signs and symptoms but no AMI‐specific codes is to identify these patients and remove obvious noncases before issuing validation questionnaires. Second, excluding codes for referral to stroke clinic or stroke monitoring increased our initial algorithm's PPV considerably but decreased its sensitivity. These codes, initially included because they seemed to indicate potential stroke in patients, did not identify incident strokes. Studies for which sensitivity is a priority should consider ways to increase the algorithm's sensitivity while retaining a reasonably high PPV. In addition, among patients electronically classified as noncases dead, 16% were found to have had an AMI or stroke. Studies estimating the incidence of AMI or stroke in this population are advised to validate all deaths of unknown cause to avoid missing these cases.

This study also found support for the use of our algorithms to identify smoking and obesity in primary care data in CPRD GOLD. Menopause is poorly captured in CPRD. For postmenopausal status, the incorporation of additional information, namely age and treatments for postmenopausal symptoms, into their definition increased the validity of the definition. The addition of two proxies for postmenopausal status resulted in a relatively small increase in the PPV and a more substantial increase in the NPV. Most importantly, the estimated prevalence of postmenopausal status increased from 24% with the original algorithm to 95% with the expanded algorithm. The latter is much closer to the expected prevalence in this population, with a mean age of 61.5 years.

The AMI and stroke IRRs closest to the best estimate were those estimated using only confirmed cases, providing evidence on the value of outcome validation. The IRR 95% CIs of all proposed outcome‐identification strategies generally overlapped. A caveat of this comparison is that the subpopulation with linkage to hospitalization and mortality data used to calculate the best estimate might differ from the subpopulation without such linkage where the validation of outcomes was performed.

A study strength was the validation of key patient characteristics that can confound epidemiologic studies. Additionally, our outcome‐identification process relied on only structured (coded) data and questionnaires that continue to be available to researchers using CPRD GOLD.

This validation study was conducted in a population of patients treated for overactive bladder because this validation effort was part of a broader study on the safety of antimuscarinic drugs.^8^, ^9^, ^15^ We do not expect that these CV outcomes and risk factors would be better or less well recorded among these patients than in the general population. Furthermore, CV risk factors investigated here might have been recorded in patients' electronic records before treatment started. For these reasons, we believe that the results presented in this paper apply to CPRD GOLD and possibly to other UK health databases with the same coding systems. Relative risks of AMI and stroke are intended to be scientifically generalizable to all patients using antimuscarinic medications to treat overactive bladder.^16^

## ETHICS STATEMENT

The study was judged to be exempt from review by the Research Triangle Institute (RTI) international institutional review board, and the study protocol was approved by the CPRD Independent Scientific Advisory Committee (protocol number 13_142A) and by the European Medicines Agency, and it was posted in the EU PAS Register in January 2014 (EU PAS Register Number: EUPAS5529).^9^

## CONFLICT OF INTEREST

Alejandro Arana, Andrea Margulis, Christine Bui, Alicia Gilsenan, Lisa McQuay, Maria Reynolds, Cristina Rebordosa, and Susana Perez‐Gutthann are employees of RTI Health Solutions, which received funding from Astellas to conduct this research. Cristina Varas‐Lorenzo was an employee of RTI Health Solutions at the time of the design and conduct of the study. Stefan de Vogel and Kwame Appenteng are employees of Astellas Pharma Global Development, the sponsors of this study. Billy Franks was an employee of Astellas Pharma Global Development at the time of the design and conduct of the study.

## REPRODUCIBILITY

The results of the study were generated by RTI Health Solutions (RTI‐HS) using data obtained from CPRD. RTI‐HS developed proprietary code to perform the analyses on the data. Researchers desiring access to the data would be required to obtain permission from the study sponsor, obtain data use agreement with CPRD, and develop their own code.

## Supporting information


**Data S1**. Microsoft excel file of code lists.

**Data S2**. PDF file of the questionnaires.

**Table S1**. Positive and negative predictive values for obesity and postmenopausal status, Microsoft word file.
