# Supplementary material for: Validation of cardiovascular outcomes and risk factors in the Clinical Practice Research Datalink in the United Kingdom
Source: Pharmacoepidemiol Drug Saf. 2020 Oct 28;30(2):237–47. doi: 10.1002/pds.5150 (PMC7821285; doi:10.1002/pds.5150)
Supplement: Supplementary file 2 — Data S2. PDF file of the questionnaires. [file PDS-30-237-s002.pdf]

RTI-HS Project # 0303528

Validation of the Clinical Practice Research Datalink for the Study of Cardiovascular Events (Astellas)

Determination of Acute Myocardial Infarction

The US Food and Drug Administration (FDA) and the European Medicines Agency (EMA) included a post-approval requirement to evaluate cardiovascular safety for a new agent to be used by patients with overactive bladder (OAB). To prepare for the post-approval evaluation, a study has been designed to validate outcome-specific case-identification algorithms based on electronic diagnosis codes in the Clinical Practice Research Datalink (CPRD). Upon validation, these algorithms will be implemented within future cohorts to evaluate cardiovascular risk associated with the new medication.

From data in the CPRD, we have identified one or more individuals in your practice with a record of having suffered an acute myocardial infarction. We request your help in confirming that these individuals represent actual cases and providing additional information on the events. We would appreciate your assistance in reviewing the medical records of these patients and completing this questionnaire for each identified patient.

We thank you in advance for your assistance. The information you provide will be very valuable in ensuring the accuracy of the results from this safety study.

CPRD practice ID: _____________________ Patient ID: ____________________________

Sex	Age at event date	Diagnosis	Date of event

Please answer all the questions listed below according the information in the written medical record on your files. Please send us de-identified copies of the necropsy report or death certificate, hospital discharge summaries, and test procedures related to this event that you might have in your files. If the patient has been transferred out of your practice or died, please complete the survey as far as you can based on your memory and tick this box if you no longer have access to the records. ☐

1. **Did the patient have an acute myocardial infarction (AMI) on or near the above recorded event date?**

   ☐ Yes

   ☐ No

   If "NO," when was the date of the most recent recorded AMI diagnosis for this

   patient? ____/______/________ (dd/mmm/yyyy)

2. **Did the patient present with a documented acute episode of cardiac arrest or symptoms consistent with myocardial ischaemia on or near the above recorded event date (symptoms might include any of the following: chest pain/pressure/ discomfort/burning, left upper extremity pain, jaw or neck pain, shortness of breath/dyspnoea, nausea/vomiting, and sweating)?**

   ☐ Yes

   ☐ No

   ☐ Unknown

   If "Yes" please confirm the date of initial symptoms

   ____/______/________ (dd/mmm/yyyy)

3. **Was the patient hospitalised for an AMI on or near the above recorded event date?**

   ☐ Yes

   ☐ No

   If "Yes," please provide the hospital admission date:

   ____/______/________ (dd/mmm/yyyy)

4. **Could you please confirm if a rise or fall of cardiac biomarkers (preferably troponins, or CPK-MB, and/or total CPK) with at least one value above the 99th percentile of the upper limit of the normal range was detected during the emergency department visit or hospitalisation?**

   ☐ Yes

   ☐ No

   ☐ No, but abnormal levels were recorded

   ☐ Levels not recorded

5. **Did the patient have an electrocardiogram (ECG) with changes indicative of new myocardial ischaemia (new ST-T changes or left bundle branch block [LBBB]) or with development of pathological Q waves?**

   ☐ Yes

   ☐ No

   ☐ ECG not recorded

6. **Did the patient have any record of imaging evidence of new loss of viable myocardium or regional wall motion abnormality or an arteriogram with recent coronary occlusion within this hospitalisation?**

   ☐ Yes

   ☐ No

   ☐ Unknown

7. **Was the patient treated with any emergent coronary revascularisation procedure (i.e., angioplasty-stent) or thrombolytic therapy for this episode?**

   ☐ Yes

   ☐ No

   ☐ Unknown

8. **Did the patient die due to an AMI or sudden cardiac death on or near the above recorded event date?**

   ☐ Yes

   ☐ No

   ☐ Unknown

   If “yes,” please tick the box that applies and provide the known date of death

   ☐ Patient died suddenly before hospital arrival

   ____/______/________ (dd/mmm/yyyy)

   ☐ Patient died in emergency department ____/______/________ (dd/mmm/yyyy)

   ☐ Patient was dead at discharge ____/______/________ (dd/mmm/yyyy)

   If a necropsy report is available for this death, was the pathology consistent with a fresh myocardial necrosis or recent coronary occlusion?

   ☐ Yes

   ☐ No

   ☐ Unknown

9. **Did the patient have a record of prior history of myocardial infarction?**

   ☐ Yes

   ☐ No

   ☐ Unknown

10. **At the time of the AMI, what was the patient’s smoking status?**

    ☐ Never smoker

    ☐ Current smoker

    ☐ Former smoker

    ☐ Unknown

11. **At the time of the AMI, was the patient considered obese (body mass index ≥ 30 kg/m^2)?**

    ☐ Yes

    ☐ No

    ☐ Unknown

12. For female patients only. At the time of the AMI, had menopause occurred?

- ☐ Yes
- ☐ No
- ☐ Unknown

RTI-HS Project # 0303528

Validation of the Clinical Practice Research Datalink for the Study of Cardiovascular Events (Astellas)

Determination of Acute Stroke

The US Food and Drug Administration (FDA) and the European Medicines Agency (EMA) included a post-approval requirement to evaluate cardiovascular safety for a new agent to be used by patients with overactive bladder (OAB). To prepare for the post-approval evaluation, a study has been designed to validate outcome-specific case-identification algorithms based on electronic diagnosis codes in the Clinical Practice Research Datalink (CPRD). Upon validation, these algorithms will be implemented within future cohorts to evaluate cardiovascular risk associated with the new medication.

From data in the Clinical Practice Research Datalink (CPRD), we have identified one or more individuals in your practice with a record of having suffered a stroke. We request your help in confirming that these individuals represent actual cases and providing additional information on the events. We would appreciate your assistance in reviewing the medical records of these patients and completing this questionnaire for each identified patient.

We thank you in advance for your assistance. The information you provide will be very valuable in ensuring the accuracy of the results from this safety study.

CPRD practice ID: ______________________ Patient ID: ______________________________

Sex	Age (at event date)	Diagnosis	Date of event

Please answer all the questions listed below according the information in the written medical record on your files. Please send us de-identified copies of the necropsy report or death certificate, hospital discharge summaries and test procedures related to this event that you might have in your files. If the patient has been transferred out of your practice or died, please complete the survey as far as you can based on your memory and tick this box if you no longer have access to the records. ☐

1. **Did the patient have an acute stroke on or near the above recorded event date?**

   ☐ Yes

   ☐ No

   If "Yes", which of the following subtypes was diagnosed

   ☐ Ischaemic

   ☐ Haemorrhagic

   ☐ Unspecified

   If "NO" when was the date of the most recent recorded stroke diagnosis for this

   patient? ____/______/________ (dd/mmm/yyyy)

2. **Did the patient present with a documented acute episode of a focal/global neurological deficit on or near the above recorded event date with at least one of the signs/symptoms consistent with stroke (e.g., change of consciousness level, hemiplegia, hemiparesis, numbness or sensory loss affecting one side of the body, dysphasia/aphasia, hemianopia, complete/partial loss of vision of one eye)?**

   ☐ Yes

   ☐ No

   ☐ Unknown

   If "Yes" please provide the date of initial symptoms

   ____/______/________ (dd/mmm/yyyy)

3. **Was the patient hospitalised for an acute stroke on or near the above recorded event date?**

☐ Yes

☐ No

If “Yes” could you please provide the hospital admission date?

____/______/________ (dd/mmm/yyyy)

If “No” was the patient referred to the neurologist due to the acute stroke?

☐ Yes

☐ No

☐ Unknown

4. **Did the patient present abnormal results of diagnostic procedures (e.g., abnormal magnetic resonance imaging of brain)?**

☐ Yes

☐ No

☐ Unknown

5. **Did the patient presented residual damage (e.g., hemiplegia, vascular dementia, aphasia) after the acute stroke episode?**

☐ Yes

☐ No

☐ Unknown

6. **Did the patient receive physiotherapy (e.g., neurological physiotherapy) for the treatment and recovery of sequelae of the stroke?**

☐ Yes

☐ No

☐ Unknown

7. **Was the patient administered acute treatment with any of the following: thrombolytic therapy or aspirin (ischaemic stroke); embolisation, clips, or other surgical procedures for aneurysm (subarachnoid haemorrhage and haemorrhagic stroke)?**
   - ☐ Yes
   - ☐ No
   - ☐ Unknown

8. **Did the patient die due to the stroke?**
   - ☐ Yes
   - ☐ No
   - ☐ Unknown

   If "yes," please tick the box that applies and provide the known date of death
   - ☐ Patient died at home or before hospital arrival

     ____/______/________ (dd/mmm/yyyy)
   - ☐ Patient died in emergency department ____/______/________ (dd/mmm/yyyy)
   - ☐ Patient was dead at discharge ____/______/________ (dd/mmm/yyyy)

   If a necropsy report is available for this death, was the pathology consistent with an acute neuronal injury?
   - ☐ Yes
   - ☐ No
   - ☐ Unknown

9. **Did the patient have a record of prior history of stroke?**
   - ☐ Yes
   - ☐ No
   - ☐ Unknown

10. **At the time of the stroke, what was the patient's smoking status?**
    - ☐ Never smoker
    - ☐ Current smoker
    - ☐ Former smoker
    - ☐ Unknown

11. At the time of the stroke, was the patient considered obese (body mass index ≥ 30 kg/m^2)?

- ☐ Yes
- ☐ No
- ☐ Unknown

12. For female patients only. At the time of the stroke, had menopause occurred?

- ☐ Yes
- ☐ No
- ☐ Unknown

RTI-HS Project # 0303528

Validation of the Clinical Practice Research Datalink for the Study of Cardiovascular Events (Astellas)

Determination of Acute Myocardial Infarction or Stroke

The US Food and Drug Administration (FDA) and the European Medicines Agency (EMA) included a post-approval requirement to evaluate cardiovascular safety for a new agent to be used by patients with overactive bladder (OAB). To prepare for the post-approval evaluation, a study has been designed to validate outcome-specific case-identification algorithms based on electronic diagnosis codes in the Clinical Practice Research Datalink (CPRD). Upon validation, these algorithms will be implemented within future cohorts to evaluate cardiovascular risk associated with the new medication.

From data in the CPRD, we have identified one or more individuals in your practice without recorded information of having suffered an acute myocardial infarction or an acute stroke. We request your help in confirming that these individuals do or do not represent actual cases and providing additional information on these types of events. We would appreciate your assistance in reviewing the medical records of these patients and completing this questionnaire for each identified patient.

We thank you in advance for your assistance. The information you provide will be very valuable in ensuring the accuracy of the results from this safety study

CPRD practice ID: ______________________ Patient ID: ______________________________

Sex	Date of study enrolment	Date of end of study follow-up

Please answer all the questions listed below according the information in the written medical record on your files. Please send us de-identified copies of the necropsy report or death certificate, hospital discharge summaries and test procedures related to this event that you might have in your files. If the patient has been transferred out of your practice or died, please complete the survey as far as you can based on your memory and tick this box if you no longer have access to the records. ☐

1. **Was the patient alive at the time of the study end date?**

   ☐ Yes

   ☐ No

2. **Did the patient have an episode of acute myocardial infarction between the study date of enrolment and the patient's end of follow-up?**

   ☐ Yes

   ☐ No

   If "Yes," please confirm the date of initial symptoms

   ____/______/________ (dd/mmm/yyyy)

3. **Did the patient have an episode of acute stroke between the study date of enrolment and the patient's end of follow-up?**

   ☐ Yes

   ☐ No

   If "Yes," please confirm the date of initial symptoms

   ____/______/________ (dd/mmm/yyyy)

   If "Yes," which of the following stroke subtypes was diagnosed?

   ☐ Ischaemic

   ☐ Haemorrhagic

   ☐ Unspecified

4. If the patient had an acute myocardial infarction (AMI), please check the recorded medical conditions that were present (check all that apply):

- ☐ Characteristic chest pain/pressure/discomfort/burning, left upper extremity pain, jaw or neck pain, shortness of breath/dyspnoea, nausea/vomiting, sweating
- ☐ Electrocardiogram (ECG) with changes indicative of new ischaemia (new ST-T changes or left bundle branch block [LBBB]) or with development of pathological Q waves
- ☐ Hospitalisation due to the AMI
- ☐ Rise or fall of cardiac biomarkers (preferably troponins or CPK-MB or total CPK) with at least one value above the 99th percentile of the upper limit of the normal range detected during the emergency department visit or hospitalisation
- ☐ Imaging evidence of new loss of viable myocardium or regional wall motion abnormality or an arteriogram with recent coronary occlusion within the hospitalisation
- ☐ Treatment with any emergent coronary revascularisation procedure or thrombolytic therapy during the acute episode

5. If the patient had an acute stroke, please check the recorded medical conditions that were present (check all that apply):

- ☐ Focal/global neurological deficit on or near the above recorded event date with at least one of the signs/symptoms consistent with stroke (e.g., change of consciousness level, hemiplegia, hemiparesis, numbness or sensory loss affecting one side of the body, dysphasia/aphasia, hemianopia, complete/partial loss of vision of one eye)
- ☐ The patient was hospitalised or referred to the neurologist due to the acute stroke
- ☐ Abnormal results of diagnostic procedures (e.g., abnormal magnetic resonance imaging of brain)
- ☐ Residual damage (e.g., hemiplegia, vascular dementia, aphasia) after the acute stroke episode
- ☐ The patient received physiotherapy (e.g., neurological physiotherapy) for the treatment and recovery of sequelae of the stroke
- ☐ The patient was treated acutely with any of the following: thrombolytic therapy or aspirin (ischaemic stroke); embolisation, clips, or other surgical procedures for aneurysm (subarachnoid haemorrhage and haemorrhagic stroke)

6. **Did the patient have a record of prior history of AMI and /or stroke?**

   AMI history

   ☐ Yes

   ☐ No

   ☐ Unknown

   Stroke history

   ☐ Yes

   ☐ No

   ☐ Unknown

7. **At the time of the study end date, what was the patient's smoking status?**

   ☐ Never smoker

   ☐ Current smoker

   ☐ Former smoker

   ☐ Unknown

8. **At the time of the study end date, was the patient considered obese (body mass index ≥ 30 kg/m^2)?**

   ☐ Yes

   ☐ No

   ☐ Unknown

9. **For female patients only. At the time of the study end date, had menopause occurred?**

   ☐ Yes

   ☐ No

   ☐ Unknown

CPRD practice ID: ____________________ Patient ID: ___________________________

RTI-HS Project # 0303528

Validation of the Clinical Practice Research Datalink for the Study of Cardiovascular Events (Astellas)

Determination of Acute Myocardial Infarction or Stroke

The US Food and Drug Administration (FDA) and the European Medicines Agency (EMA) included a post-approval requirement to evaluate cardiovascular safety for a new agent to be used by patients with overactive bladder (OAB). To prepare for the post-approval evaluation, a study has been designed to validate outcome-specific case-identification algorithms based on electronic diagnosis codes in the Clinical Practice Research Datalink (CPRD). Upon validation, these algorithms will be implemented within future cohorts to evaluate cardiovascular risk associated with the new medication.

From data in the CPRD, we have identified one or more individuals in your practice without recorded information of having an acute myocardial infarction or an acute stroke as a cause of death. We request your help in confirming that this patient has or has not suffered an AMI or stroke, providing additional information on these types of events. We would appreciate your assistance in reviewing the medical records of these patients and completing this questionnaire for each identified patient.

We thank you in advance for your assistance. The information you provide will be very valuable in ensuring the accuracy of the results from this safety study.

CPRD practice ID: ______________________ Patient ID: ______________________________

Sex	Date of enrolment	Date of death

Please answer all the questions listed below according to the information in the written medical record on your files. Please send us de-identified copies of death certificates, necropsy records, hospital discharge summaries, and test procedures related to these events that you might have in your files. As the patient has died, you may no longer have the medical records; in this situation, please complete the survey based on your memory.

1. **Did the patient have an episode of acute myocardial infarction (AMI) between the study date of enrolment and the patient's death date?**

   ☐ Yes

   ☐ No

   If "Yes" please confirm the date of initial symptoms of myocardial infarction

   ____/______/________ (dd/mmm/yyyy)

2. **Did the patient have an episode of acute stroke between the study date of enrolment and the patient's death date?**

   ☐ Yes

   ☐ No

   If "Yes" please confirm the date of initial symptoms of stroke

   ____/______/________ (dd/mmm/yyyy)

   If yes, which of the following subtypes was diagnosed?

   ☐ Ischaemic

   ☐ Haemorrhagic

   ☐ Unspecified

3. If the patient had an AMI, please check the medical conditions present (check all that apply):

- ☐ AMI was the cause of death
- ☐ Necropsy compatible with a fresh MI or recent coronary occlusion
- ☐ Characteristic chest pain/pressure/discomfort/burning, left upper extremity pain, jaw or neck pain, shortness of breath/dyspnoea, nausea/vomiting, sweating within 30 days prior to or on the date of death
- ☐ Rise or fall of cardiac biomarkers (preferably troponins or CPK-MB or total CPK) with at least one value above the 99th percentile of the upper limit of the normal range detected during the emergency department visit or hospitalisation within 30 days prior to or on the date of death
- ☐ Electrocardiogram (ECG) with changes indicative of new ischaemia (new ST-T changes or left bundle branch block [LBBB]) or with development of pathological Q waves within 30 days prior to or on the date of death
- ☐ Imaging evidence of new loss of viable myocardium or regional wall motion abnormality or an arteriogram with recent coronary occlusion in a hospitalisation within 30 days prior to or on the date of death
- ☐ Treatment with any coronary revascularisation procedure or thrombolytic therapy within 30 days prior to the or on date of death
- ☐ Hospitalisation due to the AMI within 30 days prior to or on the date of death
- ☐ Prior history of AMI or coronary heart disease

4. If the patient had an acute stroke, please check the medical conditions present (check all that apply):

- ☐ Acute stroke was the cause of death
- ☐ Necropsy consistent with an acute neuronal injury
- ☐ Focal/global neurological deficit on or near the above recorded event date with at least one of the signs/symptoms consistent with stroke (e.g., change of consciousness level, hemiplegia, hemiparesis, numbness or sensory loss affecting one side of the body, dysphasia/aphasia, hemianopia, complete/partial loss of vision of one eye) within 30 days prior to or on the date of death
- ☐ The patient was hospitalised or referred to the neurologist due to an acute stroke within 30 days prior to the date of death
- ☐ Abnormal results of diagnostic procedures (e.g., abnormal magnetic resonance imaging of brain) within 30 days prior to or on the date of death
- ☐ Residual damage (e.g., hemiplegia, vascular dementia, aphasia) after the acute stroke episode
- ☐ The patient received physiotherapy (e.g., neurological physiotherapy for the treatment and recovery of sequelae of the stroke)
- ☐ The patient was administered acute treatment: thrombolytic therapy or aspirin (ischaemic stroke); embolisation, clips, or other treatment for aneurysm (subarachnoid haemorrhage and haemorrhagic stroke) within 30 days prior to or on the date of death
- ☐ Prior history of stroke or cerebrovascular disease

5. At the time of the study end date, what was the patient's smoking status?

- ☐ Never smoker
- ☐ Current smoker
- ☐ Former smoker
- ☐ Unknown

6. At the time of the study end date, was the patient considered obese (body mass index ≥ 30 kg/m^2)?

- ☐ Yes
- ☐ No
- ☐ Unknown

7. **For female patients only. At the time of the study end date, had menopause occurred?**
   - ☐ Yes
   - ☐ No
   - ☐ Unknown
