# Supplementary material for: Validation of cardiovascular outcomes and risk factors in the Clinical Practice Research Datalink in the United Kingdom
Source: Pharmacoepidemiol Drug Saf. 2020 Oct 28;30(2):237–47. doi: 10.1002/pds.5150 (PMC7821285; doi:10.1002/pds.5150)
Supplement: Supplementary file 3 — Table S1. Positive and negative predictive values for obesity and postmenopausal status, Microsoft word file. [file PDS-30-237-s003.docx]

Table S1. Positive and Negative Predictive Values for Obesity and Postmenopausal Status

|  | CPRD Data | Questionnaires, n (%) | | |  | |
| --- | --- | --- | --- | --- | --- | --- |
|  |  | Yes | No | Unknown | PPV (95% CI) | NPV (95% CI) |
|  | **Closest date before cohort entry** | | | | | |
| Obesity: body mass index ≥ 30 kg/m^2^ | Yes | 325 (76.1) | 76 (17.8) | 26 (6.1) | 76.1 (71.8-80.1) |  |
|  | No | 33 (4.0) | 734 (90.0) | 49 (6.0) |  | 90.0 (87.7-91.9) |
|  | Unknown | 76 (16.2) | 290 (61.7) | 104 (22.1) |  |  |
| Postmenopausal status | Yes | 204 (85.7) | 7 (2.9) | 27 (11.3) | 85.7 (80.6-89.9) |  |
|  | No | 653 (76.6) | 100 (11.7) | 100 (11.7) |  | 11.7 (9.6-14.1) |
| Postmenopausal status (modified to include proxies) | Yes | 814 (91) | 16 (2) | 69 (8) | 90.5 (88.4-92.4) |  |
|  | No | 43 (22) | 91 (47) | 58 (30) |  | 47.4 (40.2-54.7) |
|  | **Closest date before the *endpoint*** | | | | | |
| Obesity: body mass index ≥ 30 kg/m^2^ | Yes | 350 (82.0) | 57 (13.3) | 20 (4.7) | 82.0 (78.0-85.5) |  |
|  | No | 27 (3.2) | 777 (91.7) | 43 (5.1) |  | 91.7 (89.7-93.5) |
|  | Unknown | 57 (13.0) | 266 (60.6) | 116 (26.4) |  |  |
| Postmenopausal status | Yes | 210 (83.3) | 9 (3.6) | 33 (13.1) | 83.3 (78.1-87.7) |  |
|  | No | 647 (77.1) | 98 (11.7) | 94 (11.2) |  | 11.7 (9.6-14.0) |
| Postmenopausal status (modified to include proxies) | Yes | 835 (90) | 19 (2) | 78 (8) | 89.6 (87.5, 91.5) |  |
|  | No | 22 (14) | 88 (55) | 49 (31) |  | 55.3 (47.3, 63.2) |
|  | **Closest date to the *endpoint* (before or after)** | | | | | |
| Obesity: body mass index ≥ 30 kg/m^2^ | Yes | 377 (83.4) | 52 (11.5) | 23 (5.1) | 83.4 (79.7-86.7) |  |
|  | No | 21 (2.2) | 862 (92.3) | 51 (5.5) |  | 92.3 (90.4-93.9) |
|  | Unknown | 36 (11.0) | 186 (56.9) | 105 (32.1) |  |  |
| Postmenopausal status | Yes | 213 (82.2) | 12 (4.6) | 34 (13.1) | 82.2 (77.0-86.7) |  |
|  | No | 644 (77.4) | 95 (11.4) | 93 (11.2) |  | 11.4 (9.3-13.8) |
| Postmenopausal status (modified to include proxies) | Yes | 837 (89) | 21 (2) | 79 (8) | 89.3 (87.2, 91.2) |  |
|  | No | 20 (13) | 86 (56) | 48 (31) |  | 55.8 (47.6, 63.8) |

AMI = acute myocardial infarction; CI = confidence interval; CPRD = Clinical Practice Research Datalink; NPV = negative predictive value; PPV = positive predictive value.

Note: the electronic algorithm identified these covariates in primary care data at three time points: closest day before cohort entry, the last date with information before the endpoint, and the date with information closest to the endpoint (before or after). General practitioners were asked to provide the same information via questionnaires on the day of the event (patients with AMI or stroke) or at study end (noncases alive or dead). Questionnaires were the gold standard for this analysis. Exact binomial CIs are reported for PPV and NPV.

Note: Postmenopausal status algorithm was modified. In addition to the codes, the updated definition included two proxies for postmenopausal status: patients aged > 50 years who used HRT therapy and patients aged > 55 years.
